# Childhood obesity in Pacific Island countries and Territories: from fragmented evidence to coordinated action

**DOI:** 10.1016/j.lanwpc.2026.101948

**Published:** 2026-07-31

**Authors:** Nicola L. Hawley

**Affiliations:** Department of Chronic Disease Epidemiology, Yale School of Public Health, USA

This three-paper series on childhood obesity in Pacific Island Countries and Territories does more than document a high-burden regional problem. By bringing together evidence on prevalence, multilevel determinants, and lifestyle interventions, the included papers provide a progression from burden, to explanation, to action. Their collective message is stark: Pacific children are experiencing among the highest levels of overweight and obesity globally, but the evidence base available to guide prevention remains fragmented, uneven, and insufficiently connected to sustained intervention.

Taken together, the three papers reveal a mismatch between the scale of childhood obesity in the Pacific and the scientific and institutional response. The prevalence estimates presented by Okely and colleagues^1^ are sufficient to establish childhood obesity as a regional priority, but not yet sufficient to guide precise, sustained, nationally actionable intervention. Novotny and colleagues usefully organize risk across socioecological levels,^2^ but also show how much more is known about individual, caregiver, and community-level correlates than about structural drivers. Finally, as Galy et al. note, the intervention evidence demonstrates creativity and commitment across several Pacific settings, but remains too thin, geographically limited, and small-scale to guide action at the level required.^3^

The gap between evidence and action matters because it affects where responsibility is located and what kinds of solutions become imaginable. With evidence concentrated around individual, caregiver, and community-level correlates,^2^ responsibility appears to sit primarily with children, families, or schools, even when the underlying drivers are broader. Diet, screen time, caregiver characteristics, school type, and physical activity are important, but they should not be overinterpreted as isolated individual-level causes. In small island settings, these factors are markers of wider social and material conditions: food imports and pricing, transport and built environments, health-system capacity, and more.^4^, ^5^, ^6^, ^7^ A child's behavior is shaped by their environment long before it appears in a survey as an individual risk factor. If the evidence base remains concentrated at lower socioecological levels, we risk mistaking what is easiest to measure for what is most important to change.

The same concern applies to intervention research. The published intervention literature described by Galy et al. is largely focused on lifestyle interventions delivered in individual, school, or community settings.^3^ These studies are important; they show that communities in the region have begun testing ways to support healthier behaviors. But their mixed and generally small effects raise a broader question: are interventions being asked to work at too narrow a level? If the drivers of childhood obesity are structural, then interventions focused primarily on knowledge, motivation, or individual behavior change are unlikely to be sufficient. The next phase of intervention research should ask not only whether a program changes BMI, diet, or physical activity, but whether it changes the conditions that make healthy growth possible.

A broader intervention agenda also requires attention to when risk emerges. Very young children are largely absent from the burden synthesis because the definitions and interpretation of overweight and obesity differ in infancy.^1^ That exclusion is methodologically understandable, but leaves a crucial prevention window underexamined. In Pacific settings where adult obesity and maternal complications of pregnancy are common,^8^^,^^9^ the first 1000 days cannot be peripheral to childhood obesity prevention. Maternal metabolic health, fetal growth, and infant feeding may all shape later obesity and cardiometabolic risk. The region also has limited longitudinal evidence linking early growth and childhood body size to later cardiovascular and metabolic risk. These pathways cannot simply be assumed from evidence generated elsewhere, particularly in a setting where obesity and cardiometabolic risk are shaped by distinctive developmental histories, environments, and genetic variation associated with body size and metabolic traits.^10^, ^11^, ^12^

The argument for filling these evidence gaps is not simply to produce a more complete epidemiologic picture; it is to make intervention more precise, timely, and effective. The region needs evidence systems designed not only to describe risk, but to act on it. The next phase of Pacific childhood obesity work should move beyond isolated prevalence studies, short-term pilots, and loosely connected programs. It should build the capacity to test, adapt, and scale interventions across settings while learning systematically from variation between countries, islands, schools, villages, and health systems. In other words, the region needs not simply more research, but a different research architecture.

Important foundations already exist. The Pacific Monitoring Alliance for NCD Action, or MANA, provides a regional accountability mechanism for monitoring NCD-related policy implementation across Pacific Island Countries and Territories.^13^^,^^14^ Pacific ECHO was established as a member-driven network for collective advocacy and action on childhood obesity,^15^ while the emerging Pacific Childhood Obesity Surveillance Initiative, P–COSI, aims to strengthen standardized child obesity surveillance. C-POND at Fiji National University is another critical asset, with a mission to generate evidence, support knowledge exchange, and build research capacity for obesity and NCD prevention.^16^ These efforts have the potential – perhaps imperative - to function not as separate initiatives, but as the building blocks of a Pacific childhood obesity learning system.

From this point forward, surveillance should be undertaken with intention. The region does not need to document childhood obesity simply for the sake of documenting it. The burden is already sufficient to justify action. A priority, therefore, should be investment in an evidence-informed Pacific childhood obesity intervention platform capable of supporting pragmatic trials, stepped-wedge evaluations, natural experiments, and hybrid effectiveness-implementation studies across multiple countries or school systems. Such a platform should not seek a single “Pacific intervention.” The diversity of the region makes that neither realistic nor desirable. Instead, the goal should be to identify which combinations of strategies are feasible, acceptable, equitable, effective, and sustainable under different conditions. Rather than importing intervention models developed elsewhere, the region can generate evidence about how prevention works when schools, families, churches, villages, health services, and regional agencies are engaged as part of a coordinated learning system. In a region of small populations, constrained resources, and shared structural pressures, shared indicators, analytic support, training, and best–practice exchange should not be considered optional additions; they are the infrastructure needed to learn at scale.

Realizing this vision requires funders to think differently. Childhood obesity prevention in the Pacific cannot be advanced through small, short-term grants that support isolated pilots while leaving coordination, implementation support, longitudinal follow-up, and regional analytic capacity unfunded. Funders should invest in the infrastructure needed for broad-scale testing and cumulative learning: data systems, implementation support teams, Pacific-led governance, and mechanisms for returning findings rapidly to communities and decision-makers.

The most important contribution of this series is that it makes visible the mismatch between the severity of childhood obesity in the Pacific and the infrastructure currently available to respond. The papers show high burden, incomplete understanding of structural drivers, limited life-course evidence, and insufficient intervention research. The next phase of work should be judged not by whether it produces another estimate of prevalence, but by whether it creates the conditions for prevention: longitudinal evidence, nationally useful data, regionally shared indicators, intervention-ready surveillance, and sustained funding for coordinated implementation science. Childhood obesity in the Pacific is a structural, intergenerational, and regional challenge that requires coordinated but locally grounded action. The evidence summarized in this series is enough to justify urgency. The task now is to build the systems needed to act with precision, evaluate with rigor, and learn at scale.

## Declaration of interests

Dr. Hawley has no conflicts of interest to declare.
